# Genomic analysis of the marine yeast *Rhodotorula sphaerocarpa* ETNP2018 reveals adaptation to the open ocean

**DOI:** 10.1186/s12864-023-09791-7

**Published:** 2023-11-20

**Authors:** Dylan M. Lane, David L. Valentine, Xuefeng Peng

**Affiliations:** 1 School of Earth, Ocean, and Environment, University of South Carolina, Columbia, SC USA; 2 Marine Science Institute, University of California, Santa Barbara, CA USA; 3 Department of Earth Science, University of California, Santa Barbara, CA USA

**Keywords:** Marine fungi, *Rhodotorula*, Genome streamlining, Yeast, Comparative genomics, Oxygen minimum zone

## Abstract

**Background:**

Despite a rising interest in the diversity and ecology of fungi in marine environments, there are few published genomes of fungi isolated from the ocean. The basidiomycetous yeast (unicellular fungus) genus *Rhodotorula* are prevalent and abundant in the open ocean, and they have been isolated from a wide range of other environments. Many of these environments are nutrient poor, such as the Antarctica and the Atacama deserts, raising the question as to how *Rhodotorula* yeasts may have adapted their metabolic strategies to optimize survival under low nutrient conditions. In order to understand their adaptive strategies in the ocean, the genome of *R. sphaerocarpa* ETNP2018 was compared to that of fourteen representative *Rhodotorula* yeasts, isolated from a variety of environments.

**Results:**

*Rhodotorula sphaerocarpa* ETNP2018, a strain isolated from the oligotrophic part of the eastern tropical North Pacific (ETNP) oxygen minimum zone (OMZ), hosts the smallest of the fifteen genomes and yet the number of protein-coding genes it possesses is on par with the other strains. Its genome exhibits a distinct reduction in genes dedicated to Major Facilitator Superfamily transporters as well as biosynthetic enzymes. However, its core metabolic pathways are fully conserved. Our research indicates that the selective pressures of the ETNP OMZ favor a streamlined genome with reduced overall biosynthetic potential balanced by a stable set of core metabolisms and an expansion of mechanisms for nutrient acquisition.

**Conclusions:**

In summary, this study offers insights into the adaptation of fungi to the oligotrophic ocean and provides valuable information for understanding the ecological roles of fungi in the ocean.

**Supplementary Information:**

The online version contains supplementary material available at 10.1186/s12864-023-09791-7.

## Background

First described over a century ago, marine fungi have received increasing recognition for their roles in ocean biogeochemical cycles and microbial food webs [1–4]. While environmental DNA sequencing surveys have uncovered many previously unrecognized lineages of marine fungi [5, 6], understanding of the functional roles of marine fungi has been partially limited by the small number of genomic analyses. Efforts such as the 1000 Fungal Genomes Project have primarily focused on fungi isolated from terrestrial environments [7]. This bias in research efforts partially stems from the view that the relative low substrate availability in seawater, especially when compared to soil and other types of terrestrial systems, limits the diversity and abundance of marine fungi.

Nutrient scarcity and the intensity of competition in oligotrophic environments is proposed to impact microbial genome size and composition. This theory, known as the streamlining theory, states that microbes recovered from environments of low nutrient availability will tend to have reduced genomes with a low base-pair count, a low intergenic-to-coding DNA ratio, few paralogs or pseudogenes, and a highly conserved set of core metabolisms [8]. Selection in oligotrophic environments is thought to favor efficiency of transport and metabolism as well as a low cost of transcription and replication [8]. To minimize the cost of protein synthesis and increase efficiency, streamlined marine microorganisms are expected to primarily reduce genes for transcriptional regulations [9]. It is supposed that carbon, nitrogen, iron, and sulfur limitations all lead to a reduction in nitrogen-rich protein expression and transition to less costly amino acid synthesis, including a preferential reduction in N-containing side chains and ribosomal proteins [10, 11]. While the streamlining theory has been supported by numerous prokaryotic examples, it is unclear whether marine fungi have made similar adaptations as their prokaryotic counterparts.

We analyzed the genome of the marine yeast *Rhodotorula sphaerocarpa* ETNP2018 isolated from the water column of the eastern tropical North Pacific (ETNP) ocean. A recent metabarcoding survey showed the genus *Rhodotorula* is one of the most prevalent and abundant fungal taxa in the ETNP oxygen minimum zone (OMZ). The *Rhodotorula* strain we analyzed was isolated from the same research expedition as the metabarcoding survey [12]. *Rhodotorula* are basidiomycetous yeasts that are sometimes referred to as “red yeasts” due to their production of β-carotene, responsible for their red, pink, or orange pigmentation [13]. Carotenogenic yeasts are utilized by the biotechnological industry to produce β-carotene as well as other isoprenoid products (γ-carotene, torulene, and torularhodin) [14]. The genus is also considered oleaginous, capable of synthesizing lipids up to one third of their dry weight. They are known to inhabit a wide array of environments, both eutrophic and oligotrophic, including human and plant hosts, benthic marine sediments, Antarctic permafrost, eutrophic freshwater lakes, ocean and river waters, soil, wood pulp, and the International Space Station [15–20]. The species *R. sphaerocarpa* has been isolated from the marine environments such as the Marguerite Bay in the Antarctic Ocean and the coastal waters of Thailand [21, 22]. In this study, we used comparative genomics of the open ocean isolate *R. sphaerocarpa* ETNP2018 and fourteen other *Rhodotorula* strains from diverse environments to test the genome streamlining theory for marine fungi and further searched for evidence of adaptation of marine fungi to the oligotrophic ocean.

## Methods

### Sampling and isolate information

The *R. sphaerocarpa* ETNP2018 strain was isolated from seawater in the eastern tropical North Pacific (ETNP) oxygen minimum zone (OMZ) [12]. Seawater from 50 m depth at Station 1 (10°N 113°W) was collected onboard R/V Sally Ride using 30-L Niskin bottles and inoculated into aerobic artificial seawater [23] medium supplemented with cellobiose (2 g L^−1^) and antibiotics (200 mg L^−1^ penicillin and streptomycin). The initial batch of enrichment cultures that showed growth were inoculated on agar plates. Axenic cultures were obtained after five passages from the first batch of agar plates. Routine maintenance of the culture was performed in 10-ml of medium at 20 °C without shaking. The strain is accessible in the Agriculture Research Service Culture Collection (Northern Regional Research Laboratory, NRRL 64474).

### Genome sequencing and assembly

Genomic DNA from 11-day cultures of *R. sphaerocarpa* ETNP2018 (OD = 0.5) was extracted using QIAGEN DNeasy Plant Mini Kit (Cat. No. 69104) following the manufacturer’s instructions. The DNA quality was checked by a NanoDrop 2000c spectrophotometer and by a Agilent TapeStation 4150 system. The DNA quantity was measured using Qubit dsDNA BR Assay Kit (Invitrogen). The DNA sample was stored at -20 °C. Sequencing libraries were constructed using the TruSeq DNA PCR Free kit (Illumina # 20015962). The draft genome of *R. sphaerocarpa* ETNP2018 was sequenced at the University of California Davis Genome Center on an Illumina HiSeq 4000 platform with 150 bp paired-end sequencing. Raw reads were filtered with the BBDuk tool in the BBMap software package (v38.73) [24] using the settings “ktrim = r ordered minlen = 51 minlenfraction = 0.33 mink = 11 tbo tpe rcomp = f k = 23 ftm = 5”. Adapters were trimmed from the BBDuk-filtered reads using the tool Trimmomatic v0.39 [25] with the settings “ILLUMINACLIP:$adapters:2:30:10 LEADING:3 TRAILING:3 SLIDINGWINDOW:4:15 MINLEN:100”. De novo assembly of reads that passed both quality filtering and adapter trimming was performed for each individual sample using SPAdes v3.15.2 [26] with kmer lengths 21, 33, 55, 77, 99, and 127.

### Phylogenomic reconstruction of Rhodotorula genomes

For phylogenomic analysis, all *Rhodotorula* genome assemblies from the National Center for Biotechnology Information’s (NCBI) Assembly Database were retrieved. The genome of *Rhodotorula sp.* FNED7-22 was excluded due to its exceptionally low quality: it contains 2,892 scaffolds with a scaffold N50 value of 5.9 kb. Five metagenome-assembled *Rhodotorula* genomes were excluded also due to similar quality issues (large number of scaffolds and small scaffold N50 values). This leaves 167 *Rhodotorula* genomes for phylogenomic analysis, as well as two genomes (*Microbotrium intermedium* GCA 900096595.1 and *Leucosporidium creatinivorum* GCA 002105055.1) selected as part of the outgroup. Benchmarking Universal Single-Copy Orthologs (BUSCO) v5.4.4 was used to retrieve single-copy orthologues from all 169 genomes [27, 28]. A total of 983 single-copy orthologues were present in all *Rhodotorula* genomes. They were aligned individually using MUSCLE v5.1 [29] and trimmed with trimAl v1.2 with the parameters “-gt 0.6 -w 3 -st 0.001” [30]. The dataset was concatenated with the perl script “catfasta2phyml.pl” which generated an accompanying partition file (https://github.com/nylander/). IQ-TREE v1.6.12 was used to find the best model for each partition and infer a maximum-likelihood tree with the parameters “-m MFP + MERGE -rcluster 10 -bb 1000 -alrt 1000” [31, 32]. All partitions shared the same set of branch lengths but are allowed to have its own evolution rate [33]. The tree was visualized using Interactive Tree Of Life v6 [34].

### Structural and functional annotations

For genome annotations and metabolic reconstruction, 14 *Rhodotorula* genomes were selected so as to capture a broad range of native habitats, as well as different species (Table S1) [16, 35–44]. BBMap was used to determine each genome’s GC content, scaffold and contiguous sequence (contig) counts, scaffold and contig N50 values, and total length [24]. Direct statistical comparison between overall averages and individual species was performed using directional one-sample t-tests at 95% confidence. Statistical analysis of differences between source categories was performed using two sample t-tests assuming unequal variance at 95% confidence.

Both structural and functional annotations were facilitated by Funannotate v1.8.14, a program for prediction, annotation, and comparison of eukaryotic genomes [45]. Each genome assembly was first soft-masked using tantan [46] and contigs < 1500 bp in length were excluded from downstream annotations. Structural annotations from BRAKER2 were generated in EPmode with OrthoDB v10 and all existing protein sequences from *Rhodotorula* genomes retrieved from the Joint Genome Institute Mycocosm as protein evidence, using ProHint as the alignment tool [7, 28, 47–49]. Because BRAKER2 utilizes GeneMark-EP + , the structural annotations from BRAKER2 are fed into the “Funannotate predict” pipeline which also uses AUGUSTUS v3.3.3 (with at least 500 training models), GlimmerHMM, and SNAP [50–52]. The SWISS-PROT protein knowledgebase, as well as existing protein sequences from *Rhodotorula* genomes retrieved from the Joint Genome Institute Mycocosm, was supplied as protein evidence to the “Funannotate predict” pipeline [7, 53]. EVidenceModeler was used to generate a weighted consensus of structural annotations from all four tools [54]. The program tRNAscan-SE was used to detect tRNA-coding genes [55].

Predicted protein sequences from structural annotations were annotated by BlastKOALA against the Kyoto Encyclopedia of Genes and Genomes (KEGG) database [56, 57], by InterProScan v5.51–85.0 [58], and by the eggnog-mapper v2.19 against the eggnog 5.0 database [59, 60]. Carbohydrate-active enzymes were identified using the dbCAN2 meta server against the Carbohydrate Enzyme (CAZy) database [61, 62]. The dbCAN2 pipeline uses three tools (DIAMOND, HMMER, and Hotpep) and we only kept annotations supported by at least two tools to be conservative and confident. To determine potential function of all well-annotated CAZymes, the CAZy family number of each reported CAZyme was compared against its entry on the online CAZypedia index [63]. Biosynthetic gene clusters (BGCs) were identified by the Antibiotics and Secondary Metabolite Analysis Shell (AntiSMASH) v6.0 using the fungal version (fungiSMASH) [64]. BGC sequences identified by AntiSMASH were submitted to the NCBI Conserved Domain Database for refined annotation of BGC genes with the location of conserved domain footprints and functional sites inferred from these footprints [65, 66].

### Metabolic reconstruction

Functional annotations were manually inspected to determine key metabolic pathways conserved in the *R. sphaerocarpa* ETNP2018 genome. Metabolic pathways were reconstructed based on the presence of key enzymes using the KEGG mapper, the *Saccharomyces* Genome Database (SGD), and published literature on budding yeasts [67–69]. Cofactors were determined using the SGD, and localization was determined using the annotations given by BlastKOALA or from the literature. This information was then used to create a custom graphic depicting the intercellular transport and localized carbohydrate metabolic pathways contained within the genome of *R*. *sphaerocarpa* ETNP2018.

## Results and discussion

### Genomic characteristics

The *R. sphaerocarpa* ETNP2018 genome was assembled into 115 scaffolds, with a total size of 17.7 Mbp, and an N50 value of 377,844 (Table 1). With 6,451 gene models, the gene density is 364 genes/Mbp, on par with previously published genomes of marine fungi [70]. BUSCO estimated that the genome is 97.3% complete. Of the 1764 BUSCOs from the OrthoDB v10 database for Basidiomycota, 1713 are present as single copies, 3 as duplicated copies, and 13 as fragmented in the *R. sphaerocarpa* ETNP2018 genome. There are 5,324 eukaryotic cluster of orthologs (KOGs), 7,872 protein family (Pfam) domains, and 137 tRNAs in the assembly. A total of 3,210 (49.8%) genes were annotated by KEGG orthology, as well as 172 CAZymes. The average genome size of all 15 *Rhodotorula* strains is 20.3 ± 1.6 Mbp and the average number of proteins per genome is 3,275 ± 154 (Table 1). The 15 *Rhodotorula* genomes included an average of 5,610 ± 360 KOG assignments, 8,240 ± 418 Pfam domains, and 194 ± 19 CAZymes. Marine yeasts isolated from seawater including *R. sphaerocarpa* ETNP2018, *R. sphaerocarpa* GDMCC 60679, *R. diobovata* 08–225, and *R. mucilaginosa* CYJ03 have an average genome size of 19.0 ± 1.6 Mbp with 3,203 ± 99 annotated proteins (Table 1).

**Table 1:** General genomic characteristics of *R. sphaerocarpa* ETNP2018 and fourteen representative *Rhodotorula* strains. "I.S.S." represents International Space Station

Source	Species	Strain	Total Length (Mbp)	GC (%)	Scaffolds	Scaffold N50 (bp)	Contigs	Contig N50 (bp)	Annotated Proteins	Gene Models	tRNA
Seawater	*R. sphaerocarpa*	ETNP2018	17.7	63.4	115	377,844	120	356,447	3210	6451	137
Seawater	*R. sphaerocarpa*	GDMCC 60679	18.0	63.0	32	1,074,774	32	1,074,774	3213	6310	316
Seawater	*R. diobovata*	08–225	21.1	67.0	361	118,648	678	82,556	3315	7741	183
Seawater	*R. mucilaginosa*	CYJ03	19.1	60.5	88	420,192	88	420,192	3073	6620	137
Marine Sediment	*R. paludigena*	P4R5	21.0	64.3	277	180,700	290	171,007	3406	7430	127
Freshwater	*R. glutinis*	ZHK	22.3	67.8	30	1,466,672	49	963,562	3376	7569	154
Freshwater	*R. kratochvilovae*	YM25235	23.7	67.3	46	1,067,950	46	1,067,950	3581	8224	294
Acid Mine Drainage	*R. taiwanensis*	MD1149	19.6	61.7	181	388,693	227	345,821	3202	6961	113
Endophytic	*R. kratochvilovae*	Y14	22.0	67.5	46	1,029,848	46	1,029,848	3465	7958	194
Endophytic	*R. graminis*	WP1	21.0	67.8	26	1,420,730	322	167,431	3213	6875	152
Terrestrial (I.S.S.)	*R. mucilaginosa*	F6_4S_B_2B	20.2	60.6	199	432,962	222	353,031	3261	7040	124
Terrestrial	*R. toruloides*	NBRC 0880	20.7	61.8	30	1,390,799	30	1,390,799	3429	8284	128
Terrestrial	*R. mucilaginosa*	B1	20.0	60.6	225	256,462	225	256,462	3256	7052	113
Terrestrial	*R. frigidialcoholis*	JG-1b	19.4	67.0	156	301,937	171	280,417	3036	6396	115
Terrestrial	*Rhodotorula sp.*	CCFEE5036	19.1	60.6	155	337,802	201	256,314	3096	6624	120
Average of all Strains			20.3	64	131	684,401	183	547,774	3275	7169	160
Stdev. of all Strains			1.6	3	103	493,511	168	425,961	154	662	64
Average of seawater strains			19	63.5	149	497,865	230	483,492	3,203	6781	193
Stdev. Of seawater strains			1.5	2.7	145	407,049	301	420,520	99	653	85

A one sample t-test found the difference in genomic size between *R. sphaerocarpa* ETNP2018 and the average of all strains to be significant (*p* = 1.059 × 10^–5^). *R. sphaerocarpa* ETNP2018’s genome is the smallest of the marine strains; however, a one sample t-test found this difference to be insignificant (*p* = 0.099). The difference was significant when compared with the mean genome size of freshwater, endophytic, and terrestrial strains (*p* = 0.04, 0.039, and 0.0008, respectively). In contrast, the number of genes with functional annotations was not different between *R. sphaerocarpa* ETNP2018 and the average of all strains, as well as marine, freshwater, endophytic, and terrestrial source category averages (*p* = 0.06, 0.44, 0.12, 0.57, and 0.24, respectively).

The genome size of *R. sphaerocarpa* ETNP2018 was 10.8% smaller than the average genome size of five representative terrestrial *Rhodotorula* strains (Table 2). This reduction of 10.8% was the largest amongst the marine strains of *Rhodotorula*, prompting the hypothesis that the environmental pressures imparted at the OMZ favor fungal strains with smaller genomes in comparison to other marine environments. This hypothesis can be tested in future studies once additional fungal genomes from the open ocean become available. A metabarcoding survey showed that *Rhodotorula* was one of the most abundant and prevalent genera of the fungal communities in the ETNP [12], indicating that *Rhodotorula* are active in the oligotrophic ocean despite their reduced genome size. The degree of genome reduction we observed in marine *Rhodotorula* strains was similar to that in the marine bacterium *Pelagibacter ubique* HTCC1062, a well-documented example of a streamlined microbe (Table 2) [71]. However, the genome reduction by the heterotrophic yeast and bacteria was not as large as the reduction (38%) found in the marine chemoautotrophic archaea *Nitrosopumilus maritimus* SCM1 when it was compared to five terrestrial strains of ammonia-oxidizing archaea (Table 2; Table S2).

**Table 2:** The percent reduction in the genomic size of *Rhodotorula* strains isolated from seawater, compared with the average of the representative terrestrial *Rhodotorula*. *Pelagibacter ubique* HTCC1062 and *Nitrosopumilis matrimis* SCM1 were chosen as well-known examples of streamlined marine microbes for comparison. *P. ubique* HTCC1062 was compared to five terrestrial strains of the alphaproteobacterial genus *Rickettsia* and *N. matrimis* SCM1 to five representative ammonia-oxidizing archaea, all retrieved from the NCBI assembly database (Table S2)

Species	Strain	Genome Size (bp)	Terrestrial Average (bp)	% Reduction
*R. sphaerocarpa*	ETNP2018	17,716,787	19,867,559	10.8
*R. sphaerocarpa*	GDMCC 60679	18,031,004	19,867,559	9.2
*R. diobovata*	08-225	21,142,622	19,867,559	no reduction
*R. mucilaginosa*	CYJ03	19,073,214	19,867,559	4.0
*Pelagibacter ubique*	HTCC1062	1,308,759	1,413,279	7.4
*Nitrosopumilus maritimus*	SCM1	1,645,259	2,670,916	38.4

The number of KOGs related to translation and ribosomal biogenesis, the transport and metabolism of amino acids, carbohydrates, lipids, secondary metabolites, and coenzymes were lower in the genome of *R. sphaerocarpa* ETNP2018 than the other 14 *Rhodotorula* strains (Tables S3 and S4) [12]. Nevertheless, *R. sphaerocarpa* ETNP2018 contained the core set of protein-coding genes despite nutrient scarcity and its small genome (Figure S1), which is consistent with streamlining in response to nutrient deprivation [8]. Previously conducted studies on streamlined microbes have found that the average genome size was the smallest for microorganisms isolated from oligotrophic seawater and the largest for those isolated from soil [8]. Microorganisms isolated from freshwater exhibit a broad spectrum of genome sizes [8]. We find *Rhodotorula* genomes from both ends of the size spectrum consistent with this theory. Among the 15 *Rhodotorula* strains, *R. glutinis* ZHK and *R. kratochvilovae* YM25235 were the largest in genome size, and they were isolated from eutrophic environments (the Pearl River and Chenghai Lake, respectively) [15, 18]. The genomes of two soil strains, *R. frigidialcoholis* JG-1b and *R. sp.* CCFEE5036, were smaller than the average genome size of all 15 strains. However, both of these strains were isolated from permafrost and hyper-arid soil in Antarctica’s McMurdo Dry Valley [37, 39]. Their reduced genome size could be related to the extreme conditions of their environment with low nutrient availability.

### Phylogeny

**Fig. 1:**
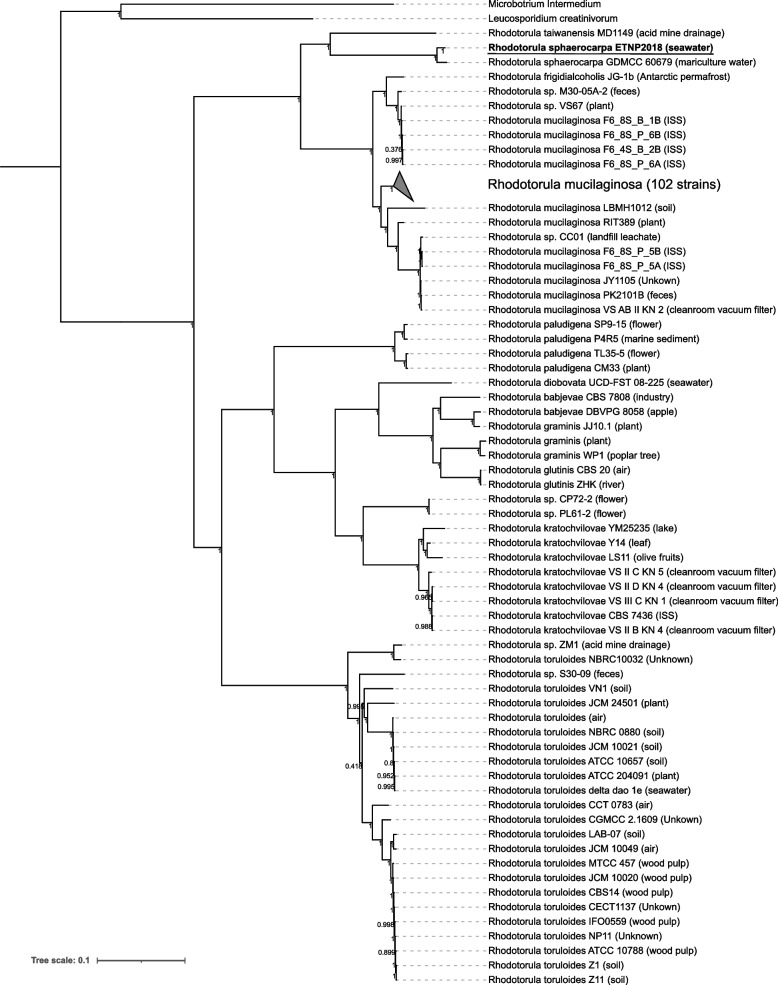
A phylogenomic tree constructed using single-copy orthologues shared amongst 168 *Rhodotorula* strains (as well as two outgroup species, *Microbotrium intermedium* GCA 900096595.1 and *Leucosporidium creatinivorum* GCA 002105055.1). 983 single-copy orthologs were retrieved for comparison using BUSCO v5.4.4. The isolation source is denoted in the parentheses after the strain names (“ISS” = International Space Station). Bootstrap values (*n* = 1000) are displayed at each node. The collapsed clade includes 102 strains of *Rhodotorula mucilaginosa* primarily from the International Space Station. The two side lengths of the triangle representing the collapsed clade are proportional to the distances to the node’s closest and farthest child leaves. *R. sphaerocarpa* ETNP2018 is highlighted by bold font and an underline. The same tree that displays the 102 collapsed *Rhodotorula mucilaginosa* strains is included as Figure S2

Phylogenomic analysis revealed multiple monophyletic clades at the level of species, including *R. sphaerocarpa*, *R. paludigena*, *R. kratochvilovae*, and *R. toruloides* (Fig. 1 and Figure S2). *R. sphaerocarpa* ETNP2018 was closely related to the *R. sphaerocarpa* strain isolated from mariculture seawater in Maoming, Guangdong, China. Together, these two *R. sphaerocarpa* genomes form a clade distinct from all other *Rhodotorula* species, demonstrating the evolutionary pressure that likely contributed to the speciation of *R. sphaerocarpa*, which has been primarily isolated from seawater [22, 72–74]. Among the *Rhodotorula,* some strains of the same species have been isolated from drastically different environments (Fig. 1). This could be attributed to the ability of *Rhodotorula* yeasts to adapt to a diverse range of environmental conditions [14, 39]. *R. mucilaginosa*, for example, has been isolated from soil, both animal and plant microbiomes, industrial mineral deposits, the International Space Station, and the marine water column [19, 43]. Our phylogenomic reconstruction shows that the recently described *R. frigidialcoholis* JG-1b [20] is closely related to *R. mucilaginosa* (Fig. 1). The sister clade of the main *R. mucilaginosa* clade includes *R. frigidialcoholis* JG-1b, two genomes of undescribed species, and four *R. mucilaginosa* genomes, so it is possible that these four *R. mucilaginosa* genomes obtained from the international space station may have been misclassified and are actually *R. frigidialcoholis*.

### CAZymes

**Fig. 2:**
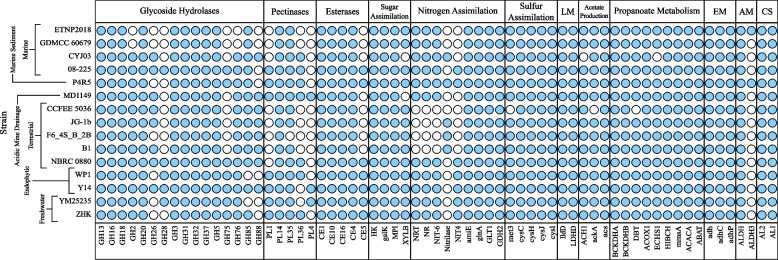
Heatmap of major genes involved in carbohydrate, nitrogen, and sulfur metabolisms in the *Rhodotorula* genome. Filled circle (light blue) indicate the presence of a gene whereas empty circles indicate its absence. Abbreviations for the headers are as follows: LM, Lactate Metabolism; EM, Ethanol Metabolism; AM, Aldehyde Metabolism; CS, Carotenoid Synthesis. The described functions of glycoside hydrolases (GH), pectinases (PL) and esterases (CE) are in Table S6. Abbreviations for the gene names are as follows: HK, hexokinase; galK, galactokinase; MPI, mannose-6-phosphate isomerase; XYLB, xylulokinase; NRT, nitrate/nitrite transporter; NR, nitrate reductase (NAD(P)H); NIT-6, nitrite reductase (NAD(P)H); NIT4, nitrate assimilation; amiE, aliphatic amidase; glnA, glutamine synthetase; GLT1, glutamate synthase (NADH); GDH2, glutamate dehydrogenase; met3, sulfate adenylyltransferase; cysC, adenylylsulfate kinase; cysH, phosphoadenosine phosphosulfate reductase; cysJ, sulfite reductase (NADPH) flavoprotein alpha-component; cysI, sulfite reductase (NADPH) hemoprotein beta-component; lldD, L-lactate dehydrogenase; LDHD, D-lactate dehydrogenase; ACH1, acetyl-CoA hydrolase; ackA, acetate kinase; acs, acetyl-CoA synthetase; BCKDHA, 2-oxoisovalerate dehydrogenase alpha subunit; BCKDHB, 2-oxoisovalerate dehydrogenase beta subunit; DBT, dihydrolipoyl transacylase; ACOX1 acyl-CoA oxidase; ECHS1, enoyl-CoA hydratase; HIBCH, 3-hydroxyisobutyryl-CoA hydrolase; mmsA, acetyl-CoA carboxylase; ACACA, malonate-semialdehyde dehydrogenase; ABAT, 4-aminobutyrate aminotransferase; adh, alcohol dehydrogenase (NADP +); adhC, alcohol dehydrogenase; adhP, alcohol dehydrogenase, propanol-preferring; ALDH, aldehyde dehydrogenase (NAD +); ALDH3, aldehyde dehydrogenase (NAD(P) +); AL2, 15-cis-phytoene synthase / lycopene beta-cyclase; AL1, phytoene desaturase

CAZymes identified in the *R. sphaerocarpa* ETNP2018 genome include 56 glycoside hydrolases (GH), 84 glycosyltransferases (GT), 17 related to auxiliary activities (AA), 3 carbohydrate binding modules (CBM), 8 carbohydrate esterases (CE), and 4 polysaccharide lyases (PL) (Table S5). Chitinase (GH18), xyloglucanase (GH16), β-hexosaminidase (GH20), both α- and β-glucosidase (GH3/31), invertase (GH32), α,α-trehalase (GH37), α-mannosidase (GH38/47), and cellulase (GH5) glycoside hydrolase CAZymes are all conserved across the *Rhodotorula* genus (Fig. 2). Therefore, *Rhodotorula* yeasts have the potential to digest chitin, xylan, hexoses, trehalose, some mannose, and cellulose [63]. Trehalase and glycogen debranching CAZymes (GH13) are also present in all 15 representative members of the *Rhodotorula* genus, suggesting the ability to use both storage polysaccharides to maintain energy production in response to potential carbon deprivation (Fig. 2). The most prevalent and abundant glycosyl hydrolase families include GH5, GH16, and GH18 (Figure S3).

Chitin is the most abundant biopolymer found in the marine environments and thus an important source of carbon and nitrogen for marine microbes [75]. Chitin is produced throughout the water column by fungi, protists, and crustaceans, yet is utilized so rapidly that it is present only in trace concentrations in marine sediments. The importance of chitin as a source of nutrient for marine fungi is relatively understudied however, as most marine chitin degradation is attributed to bacteria [75]. Chitinase is conserved throughout the *Rhodotorula* genus. GH18 was the most prevalent in genomes of freshwater strains (7 ± 1.4) and the least in genomes of marine strains (4 ± 1.4). Chitin degradation via chitinase results in a wide array of oligomers including diacetylchitobiose. The endo-β-N-acetylglucosaminidase (GH85, EC: 3.2.1.96), which degrades diacetylchitobiose into monomeric residues of β-1,4-N-acetyl-D-glucosamine (GlcNAc) and subsequently soluble sugars and dissolved organic nitrogen, was present in all *Rhodotorula* genomes except for *R. graminis* WP1 and *R. kratochvilovae* YM25235 (Fig. 2) [75, 76].

GH2, a β-mannosidase, and GH76, an α-1,6-mannanase, are absent exclusively in the two genomes of *R. sphaerocarpa* strains isolated from seawater (Fig. 2). Polygalacturonase (GH28), β-glucoronyl hydrolase (GH88), chitonsanase (GH75), and β-mannanase (GH26) CAZymes are absent in *R. sphaerocarpa* ETNP2018 as well as several other strains (Fig. 2). Mannanase and mannosidase allow yeast to ferment mannose to ethanol [77]; reduction in mannose-hydrolyzing CAZymes in the *R. sphaerocarpa* ETNP2018 genome suggests that it is not a commonly utilized substrate for the strain. Mannans are typically found in plant vacuoles and the endosperm of seeds, as well as the cell walls of certain yeasts [77]. These sources place open ocean yeast such as *R. sphaerocarpa* ETNP2018 far from a consistent supply of mannans, suggesting that its reduction in mannanase and mannosidase CAZymes is a response to the low encounter frequency for the substrate [77, 78].

A recent study demonstrated a positive correlation between a fungal strain’s repertoire of CAZymes and its saprophytic tendencies [79]. This suggests that strains which encode few CAZymes, such as those isolated from the water column, have less saprophytic tendencies and encounter fewer carbohydrates than those with higher counts, such as freshwater or endophytic strains. Fungi are the dominant detritovores in eutrophic freshwater ecosystems, and endophytic fungi are reported to opportunistically utilize saprophytic feeding mechanisms after the death of their host plant [80, 81]. The availability of organic matter in these environments makes the synthesis of many different CAZymes more energetically favorable in comparison with the oligotrophic open ocean. Given the low availability of organic matter in the open ocean water column, *R. sphaerocarpa* ETNP2018 likely streamlined its genome to reduce unnecessary and biosynthetically expensive CAZymes.

### Central carbon metabolisms

**Fig. 3:**
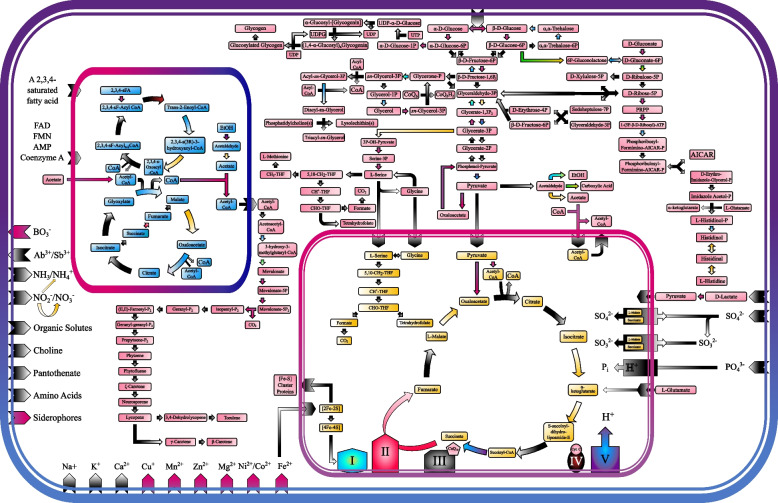
Reconstructed metabolic pathways for *Rhodotorula sphaerocarpa* ETNP2018. The outer bi-layered membrane represents plasma membrane surrounding the cytosol, the inner bi-layered membrane the mitochondrion, and the inner single-layered membrane the peroxisome; shapes embedded into membranes represent transport proteins. Arrow and transport protein color gradients represent requirement for a specific cofactor in reaction or transport, with the second color of the gradient indicating preferred direction: Black/White, no cofactor required; Light Blue/White, H_2_O; Purple/Pink, ATP; Purple/Blue, ADP; Yellow/White, NAD^+^; Teal/Yellow, NADH; Green/Yellow, NADP^+^; Green/White, NADPH; Red/Pink, FAD^2+^; Black/Red, O_2_; Purple/Red, H_2_O_2_. Abbreviations are as follows: EtOH, Ethanol; CoA, Coenzyme A; THF, tetrahydrofolate; P_i_, Inorganic Phosphate; AICAR, 5-aminoimidazole-4-carboxamide-1-β-D-ribofuranoside; PRPP, phosphoribosyl diphosphate; AMP, adenosine monophosphate; ATP, adenosine triphosphate; UDP, uridine diphosphate; UTP, uridine triphosphate; UDPG, UDP-α -D-Glucose; sFA, Saturated Fatty Acid; FAD, Flavin Adenine Dinucleotide; FMN, Flavin Mononucleotide; CoQ_6_, Ubiquinone-6; CoQ_6_H_2_, Ubiquinol-6; CoQ_10_, Ubiquinone-10; Cyt. C, Cytochrome C

The Embden-Meyerhof-Parnas pathway, Tricarboxylic acid (TCA) cycle, glyoxylate cycle, and pentose phosphate pathway were present in their entirety in the *R. sphaerocarpa* ETNP2018 genome (Figure S1). Potential substrates for these pathways include glucose, the cell’s preferred substrate, as well as acetate, ethanol, D-lactate, L-glutamine, and oxaloacetate (Fig. 3). The glyoxylate cycle, a secondary shunt of the TCA cycle localized in the peroxisome, utilizes isocitrate lyase (EC: 4.1.3.1) to catalyze the conversion of isocitrate to glyoxylate as well as succinate to malate without requiring the energy intensive decarboxylation steps required to form S-succinyl-dihydrolipoamide-E from isocitrate during the TCA cycle (Fig. 2) [82]. Glyoxylate cycle genes in yeast have been shown to upregulate in macrophage-engulfed *Candida* yeasts, concurrent with a downregulation of transcriptional machinery and glycolytic enzymes, allowing the cell to acquire carbon through alternative sources to glucose and conserve energy [82]. This suggests preferential use of the glyoxylate cycle as a response to glucose deprivation which is typical in the oligotrophic ocean.

In case physical processes transport *R. sphaerocarpa* ETNP2018 to the anoxic part of the water column, its genome shows the potential to ferment pyruvate via the enzyme pyruvate decarboxylase (EC: 4.1.1.1), creating acetaldehyde that can be further converted to acetate, ethanol, or carboxylic acids (Fig. 2). Acetate is synthesized from acetaldehyde via aldehyde dehydrogenase (EC: 1.2.3.1) to replenish acetyl-CoA using its acetyl group (Fig. 2). Ethanol is then synthesized via alcohol dehydrogenase (EC: 1.1.1.2) alongside the interconversion of NADH to NAD^+^ as a mechanism of replenishing the intracellular reducing agent (Figs. 2 and 3). It can then be excreted passively or converted to acetate in the peroxisome (Fig. 3). The small number of Pfam domains related to short chain dehydrogenase enzymes (PF00106), which are responsible for fermentative reactions on aldehydes and alcohols (Table 3), suggests the niche of *R. sphaerocarpa* ETNP2018 is not the anoxic portion of the water column. Reduced fermentative machinery may rather serve as an adaptation to their oligotrophic yet oxygenated environment, where biosynthetic resources are at a premium, and anaerobic metabolisms act as a backup for unfavorable changes in conditions.

**Table 3:** Pfam domains with major depletions in the genome of *R*. *sphaerocarpa* ETNP2018 as compared to the average of all *Rhodotorula* strains studied

Domain ID	Description	*R. sphaerocarpa* ETNP2018	Average	Standard Deviation
PF07690	Major Facilitator Superfamily	101	129.7	19.0
PF00172	Fungal Zn(2)-Cys(6) binuclear cluster domain	49	55.7	11.6
PF00106	Short Chain Dehydrogenase	37	45.3	6.1
PF13561	Enoyl-(Acyl carrier protein) reductase	35	44.4	6.0
PF08659	KR Domain	30	36.9	5.4
PF04082	Fungal specific transcription factor domain	21	29.7	4.0
PF12937	F-box-like	20	34.6	13.1
PF00646	F-box domain	14	22.2	4.8
PF01753	MYND finger	7	24.7	15.1
PF03171	2OG-Fe(II) oxygenase superfamily	5	9.6	3.4
PF02668	Taurine catabolism dioxygenase TauD, TfdA family	5	9.9	3.6
PF01179	Copper amine oxidase, enzyme domain	0	2.1	1.0
PF02727	Copper amine oxidase, N2 domain	0	1.1	0.7
PF03452	ANP1 (alpha-1,2-mannosyltransferase)	0	1.7	0.7
PF02194	PXA domain	0	1.7	0.7
PF07683	Cobalamin synthesis protein cobW C-terminal domain	0	1.7	0.6

### Transporters

Compared to other *Rhodotorula* strains, the genomes of both *R. sphaerocarpa* ETNP2018 and GDMCC 60679 are particularly low in the number of the Pfam domain encoding the major facilitator superfamily (MFS) of transporters (PF07690) (Table 3), which play a significant role in the cross-membrane transport of organic solutes. This suggests *R. sphaerocarpa* strains isolated from seawater have streamlined their genomes given the low substrate availability in the ocean. MFS transporters also symport H^+^ with siderophores, organometallic molecules formed by prokaryotes to sequester ferric iron [83, 84]. Although yeasts are considered to utilize siderophore assimilation as an opportunistic iron uptake mechanism [84], the low number of MSF transporters in the genomes of *R. sphaerocarpa* strains suggests a low competitiveness in siderophore acquisition, a tradeoff resulting from genome streamlining.

Nevertheless, the number of other metal transporters annotated by Pfam was not lower in the genomes of *R. sphaerocarpa* ETNP2018 and GDMCC 60679 in comparison to the other strains. High affinity iron permease (PF03239) was conserved in all 15 *Rhodotorula* genomes, suggesting ferrous iron intake provides them with much of the iron required for protein synthesis. One copy of PF10566, which contains natural resistance associated macrophage protein (Nramp) transporters, was conserved across all 15 *Rhodotorula* strains, aside from *R. toruloides* NBRC 0880, which contains two, and *R. kratochvilovae* YM25235, which contains zero. Nramp transporters, belonging to the Smf family of genes, are responsible for the cross-membrane transport of a variety of transition metals [85, 86]. Smf proteins demonstrate the highest affinity for Cu^2+^ and Mn^2+^ and are thought to be responsible for the high-affinity Mn^2+^ uptake system, but also show function in transporting ferrous iron, copper, nickel, cadmium, cobalt, zinc, and manganese [83, 85].

### Nitrogen assimilation

Ten of the 15 representative *Rhodotorula* strains encode the genes for nitrate assimilation pathways (Fig. 2), through which nitrate and nitrite are transported into the cell by the nitrate/nitrite transporter narK and reduced to ammonium via the enzymes nitrate reductase (EC: 1.7.1.1) and nitrite reductase (EC: 1.7.1.4) (Fig. 3). Six of these ten genomes were isolated from aquatic sources: two from freshwater, *R. glutinis* ZHK and *R. kratochvilovae* YM25235, and four from the marine environment, *R. sphaerocarpa* ETNP2018, *R. paludigena* P4R5, *R. sphaerocarpa* GDMCC 60679, and *R. diobovata* 08–225 (Fig. 2). The only aquatic yeast lacking this genetic potential is *R. mucilaginosa* CYJ03, isolated from the Yellow Sea in Yunnan, China (Fig. 2) [43].

Nitrate assimilation, in particular the reduction of nitrate in the cytosol, is an energetically expensive process [87]. Strains lacking the genetic potential to assimilate nitrate were largely isolated from environments where competition for resources is less intense and alternative sources of nitrogen (e.g. ammonium, urea) are likely readily available. *R. mucilaginosa* CYJ03 was isolated from the northern Yellow Sea, which has been eutrophic for decades [88]. It can therefore be inferred that *R. mucilaginosa* CYJ03 encounters comparatively high ammonium concentrations in the water column and to synthesize nitrate reductase would constitute a waste of biosynthetic resources.

Yeasts in the genus *Rhodotorula* have previously displayed the ability to grow on acetonitrile as a sole nitrogen source [89]. Nitrile hydratase (NHase) proteins, together with amidases (EC: 3.5.1.4), mediate a two-step metabolism of nitrile compounds such as acetonitrile to amides and acids; a second nitrile-hydrolyzing enzyme found in yeast, nitrilase (EC: 3.5.5.1), can perform the same reaction in one step. One of either nitrilase or cyanoalanine nitrilase (EC: 3.5.5.4) was found in all *Rhodotorula* genomes except for the two *R. sphaerocarpa* strains, *Rhodotorula sp*. CCFEE5036, and *R. taiwanensis* MD1149 (Fig. 2). However, none of the representative strains contained genes for NHase synthesis. Acetonitriles are predominately released via terrestrial biomass burning and constitute only a trace gas in the global atmosphere, placing open ocean *R. sphaerocarpa* strains far from stable sources of incorporable nitriles [90]. The lack of NHase, *CobW,* and nitrilase genes exclusively in both *R. sphaerocarpa* ETNP2018 and *R. sphaerocarpa* GDMCC 60679 suggests that as the lineage was diverging, *R. sphaerocarpa* strains did not retain *CobW* or nitrilase genes potentially due to a lack of available nitrile compounds in the ocean.

### Secondary metabolisms

All *Rhodotorula* genomes contained between four and six BGCs, primarily from the categories of non-ribosomal polyketide synthase (NRPS) and Terpene synthesis. The genome of *R. sphaerocarpa* ETNP2018 included NRPS-like clusters 1.1 and 6.1 as well as terpene clusters 8.1 and 9.1. The core biosynthetic gene of terpene cluster 9.1 functions in the formation of an isoprenoid biosynthetic complex. A BLASTp search identified both lycopene β-cyclase (EC: 5.5.1.19) and phytoene synthase (EC: 2.5.1.32) domains in the complex. Both lycopene and phytoene result from the digestion of cytosolic acetyl-CoA during the mevalonate (MVA) pathway, which converts acetyl-CoA into isopentyl diphosphate (Fig. 3) [91]. Phytoene is converted to lycopene via the enzymatic action of phytoene desaturase (EC: 1.3.99.30), where it can be further metabolized to form the carotenoid β-carotene via lycopene β-cyclase [91]. Yeast carotenoids are responsible for protection from over-exposure to ultraviolet light, in addition to proposed antimicrobial activity [92]. *Rhodotorula* have been shown to increase carotenogenesis as light intensity increases, indicating the molecules have a photoprotective role in the cell [92, 93]. *R. sphaerocarpa* ETNP2018 encodes five laccases (AA1), the most of all 15 *Rhodotorula* analyzed (Table S6). Fungal laccases can be ligninolytic enzymes, and are also known to function in plant pathogenicity, detoxification, and pigment modification [94]. Laccases degrade β-carotene and other carotenoids, so they may play a role in the breakdown of intracellular carotenoid pigments. Light is attenuated more rapidly in eutrophic lakes than in pelagic seawater due to the presence of particulate and dissolved organic matter, so lake yeasts would have less exposure to potentially harmful UV light and thus a reduced requirement for pigmented molecules [95].

## Conclusions

Our analysis suggests that the marine yeast *R. sphaerocarpa* ETNP2018 has adapted to conditions in the oligotrophic marine environment through genomic and subsequent biosynthetic streamlining. Its genome is smaller than the typical *Rhodotorula* strain, allowing it to conserve limited nutrients during replication without a massive reduction in potential proteins. Reduction was expected for transcription-related genes but was primarily found in biosynthetic genes. In the genome of *R*. *sphaerocarpa* ETNP2018, the number of KOGs related to carbohydrate, lipid, and secondary metabolisms was lower than average, and depleted Pfam domains were mostly related to transport and non-essential biosynthetic pathways. The number of CAZymes was lower in the genome of *R*. *sphaerocarpa* ETNP2018 than the average of *Rhodotorula* genomes, and the reduction in CAZymes was primarily found among CAZymes involved in the metabolism of non-marine biopolymers. The conservation of core carbohydrate metabolisms in the genome of *R*. *sphaerocarpa* ETNP2018, including carotenoid production, suggests it has maintained an independent lifestyle despite streamlining pressures.

### Supplementary Information

**Additional file 1: Table S1.** The NCBI accession number, origin, and isolation source/host of *Rhodotorula* genomes chosen for comparison. **Table S2.** Bacterial and archaeal genomes selected for comparison to *Pelagibacter ubique* HTCC1062 and *Nitrosopumilus maritimus* SCM1. **Table S3.** Eukaryotic clusters of orthologous groups (KOGs) assigned to each functional category for fifteen *Rhodotorula *species. **Table S4.** Statistical analysis of the depletion of specific KOG categories in *Rhodotorula sphaerocarpa* ETNP2018, performed via one sample T-test. All tests were performed using a 95% confidence interval and the average of all fifteen representative *Rhodotorula* strains. **Table S5.** CAZyme families present in 15 representative Rhodotorula strains determined using dbCAN2. The carbohydrate binding module domains (CBM) column only includes genes containing both glycoside hydrolase (GH) and carbohydrate binding module domains (CBM). **Table S6.** All CAZymes (including their described functions) present in 15 representative Rhodotorula strains. **Figure S1.** A heatmap showing highly conserved carbohydrate metabolism pathways in the *Rhodotorula *genus. **Figure S2.** A phylogenomic tree constructed using single-copy orthologues shared amongst 168 representative *Rhodotorula *strains (as well as two outgroup species, *Microbotrium intermedium* GCA 900096595.1 and *Leucosporidium creatinivorum* GCA 002105055.1). **Figure S3.** Heatmap showing the number of major CAZymes present in the *Rhodotorula *genus.

## Data Availability

The genomic data for *R. sphaerocarpa* ETNP2018 is available at the NCBI with the BioSample number SAMN15201391.
