# Supplementary material for: Genomic analysis of the marine yeast Rhodotorula sphaerocarpa ETNP2018 reveals adaptation to the open ocean
Source: BMC Genomics. 2023 Nov 20;24:695. doi: 10.1186/s12864-023-09791-7 (PMC10662464; doi:10.1186/s12864-023-09791-7)
Supplement: Supplementary file 1 — Additional file 1: Table S1. The NCBI accession number, origin, and isolation source/host of Rhodotorula genomes chosen for comparison. Table S2. Bacterial and archaeal genomes selected for comparison to Pelagibacter ubique HTCC1062 and Nitrosopumilus maritimus SCM1. Table S3. Eukaryotic clusters of orthologous groups (KOGs) assigned to each functional category for fifteen Rhodotorula species. Table S4. Statistical analysis of the depletion of specific KOG categories in Rhodotorula sphaerocarpa ETNP2018, performed via one sample T-test. All tests were performed using a 95% confidence interval and the average of all fifteen representative Rhodotorula strains. Table S5. CAZyme families present in 15 representative Rhodotorula strains determined using dbCAN2. The carbohydrate binding module domains (CBM) column only includes genes containing both glycoside hydrolase (GH) and carbohydrate binding module domains (CBM). Table S6. All CAZymes (including their described functions) present in 15 representative Rhodotorula strains. Figure S1. A heatmap showing highly conserved carbohydrate metabolism pathways in the Rhodotorula genus. Figure S2. A phylogenomic tree constructed using single-copy orthologues shared amongst 168 representative Rhodotorula strains (as well as two outgroup species, Microbotrium intermedium GCA 900096595.1 and Leucosporidium creatinivorum GCA 002105055.1). Figure S3. Heatmap showing the number of major CAZymes present in the Rhodotorula genus. [file 12864_2023_9791_MOESM1_ESM.docx]

# Supplementary Information for

**Genomic analysis of the marine yeast *Rhodotorula sphaerocarpa* ETNP2018 reveals adaptation to the open ocean**

### Dylan M. Lane^a^, David L. Valentine^b,c^, Xuefeng Peng^a,b#^

^a^School of Earth, Ocean, and Environment, University of South Carolina, Columbia, SC, USA

^b^Marine Science Institute, University of California, Santa Barbara, CA, USA

^c^Department of Earth Science, University of California, Santa Barbara, CA, USA

#Corresponding Author (email: [xpeng@seoe.sc.edu](mailto:xpeng@seoe.sc.edu))

## Table of contents

## Tables

**Table S1**. The NCBI accession number, origin, and isolation source/host of *Rhodotorula* genomes chosen for comparison.

**Table S2**. Bacterial and archaeal genomes selected for comparison to *Pelagibacter ubique* HTCC1062 and *Nitrosopumilus maritimus* SCM1.

**Table S3**. Eukaryotic clusters of orthologous groups (KOGs) assigned to each functional category for fifteen *Rhodotorula* species.

**Table S4**. Statistical analysis of the depletion of specific KOG categories in *Rhodotorula sphaerocarpa* ETNP2018, performed via one sample T-test. All tests were performed using a 95% confidence interval and the average of all fifteen representative *Rhodotorula* strains.

**Table S5**. CAZyme families present in 15 representative *Rhodotorula* strains determined using dbCAN2. The carbohydrate binding module domains (CBM) column only includes genes containing both glycoside hydrolase (GH) and carbohydrate binding module domains (CBM).

**Table S6**. All CAZymes (including their described functions) present in 15 representative *Rhodotorula* strains.

## Figures

**Figure S1**. A heatmap showing highly conserved carbohydrate metabolism pathways in the *Rhodotorula* genus. Genes present in the genome are represented by filled circles; genes absent in the genome are represented by empty circles. Abbreviations for the gene names are as follows: GPI, glucose-6-phosphate isomerase; PFK, 6-phosphofructokinase 1; FBA, fructose-bisphosphate aldolase, class II; GAPDH, glyceraldehyde 3-phosphate dehydrogenase; PGK, phosphoglycerate kinase; PGAM, 2,3-bisphosphoglycerate-dependent phosphoglycerate mutase; ENO, enolase; PK, pyruvate kinase; FBP, fructose-1,6-bisphosphatase I; PDHA, pyruvate dehydrogenase E1 component alpha subunit; PDHB, pyruvate dehydrogenase E1 component beta subunit; CS, citrate synthase; ACO, aconitate hydratase; IDH1, isocitrate dehydrogenase; IDH3, isocitrate dehydrogenase (NAD+); OGDH, 2-oxoglutarate dehydrogenase; DLST, dihydrolipoamide succinyltransferase; LSC1, succinyl-CoA synthetase alpha subunit; LSC2, succinyl-CoA synthetase beta subunit; SDHA, succinate dehydrogenase flavoprotein subunit; SDHB, succinate dehydrogenase iron-sulfur subunit; SDHC, succinate dehydrogenase cytochrome B560 subunit; SDHD, succinate dehydrogenase membrane anchor subunit; FH, fumarate hydratase; MDH2, malate dehydrogenase; pckA, phophoenolpyruvate carboxykinase (ATP); PC, pyruvate carboxylase; aceA, isocitrate lyase; aceB, malate synthase; katE, catalase; idnK, gluconokinase; PGD, 6-phosphogluconate dehydrogenase; rpiA, ribose 5-phosphate pyrophosphokinase; PRPS, ribose-phosphate pyrophosphokinase; rbsK, ribokinase; pgm, phosphoglucomutase; rpe, ribulose-phosphate 3-epimerase; tktA/tktB, transketolase; talA/talB, transaldolase; xfp, xylulose-5-phosphate/fructose-6-phosphate phosphoketolase; xpk, fructose-6-phosphate phophoketolase; G6PD, glucose-6-phosphate 1-dehydrogenase; PGLS, 6-phosphogluconolactonase.

**Figure S2**. A phylogenomic tree constructed using single-copy orthologues shared amongst 168 representative *Rhodotorula* strains (as well as two outgroup species, *Microbotrium intermedium* GCA 900096595.1 and *Leucosporidium creatinivorum* GCA 002105055.1). 983 single-copy orthologs were retrieved for comparison using BUSCO v5.4.4. The isolation source is denoted in the parentheses after the strain names (“ISS” = International Space Station). Bootstrap values (n = 1000) are displayed at each node. *R. sphaerocarpa* ETNP2018 is highlighted by a bold blue font.

**Figure S3**. Heatmap showing the number of major CAZymes present in the *Rhodotorula* genus.

**Table S1**. The NCBI accession number, origin, and isolation source/host of *Rhodotorula* genomes chosen for comparison.

| **Environment** | ***Rhodotorula* Species** | **Strain** | **NCBI Accession Number** | **Origin** | **Source/Host** |
| --- | --- | --- | --- | --- | --- |
| Marine | *sphaerocarpa* | ETNP2018 | GCA_030983975.1 | Eastern Tropical North Pacific | ETNP Oxygen minimum zone (Euphotic Zone) |
| Marine | *sphaerocarpa* | GDMCC 60679 | ASM2471353v1 | Maoming, Guangdong Province, China | Mariculture Water (Seawater) |
| Marine | *diobovata* | 08-225 | GCA_006352295.1 | Southern Florida, USA | Coastal Seawater |
| Marine | *paludigena* | P4R5 | ASM1967111v1 | South Pacific | Deep Ocean Sediment (4067 m) |
| Marine | *mucilaginosa* | CYJ03 | ASM414367v1 | Qingdao, China | Yellow Sea |
| Freshwater | *kratochvilovae* | YM25235 | ASM1992602v1 | Yunnan, China | Chenghai Lake (eutrophic) |
| Freshwater | *glutinis* | ZHK | ASM1550198v1 | Pearl River, China | Pearl River (subsurface) |
| Drainage | *taiwanensis* | MD1149 | ASM292249v1 | Maryland, USA | Acid Mine Drainage Sediment |
| Endophytic | *graminis* | WP1 | GCF_001329695.1 | Washington, USA | *P. trichocarpa* (California poplar) |
| Endophytic | *kratochvilovae* | Y14 | ASM2281782v1 | Tübingen, Germany | *A. thaliana* (mouse-ear cress) |
| Terrestrial | *mucilaginosa* | F6_4S_B_2B | ASM1919093v1 | International Space Station (USA) | Aluminum/Stainless Steel Surface |
| Terrestrial | *mucilaginosa* | B1 | ASM1868784v1 | Guangxi, China | Soil |
| Terrestrial | *frigidialcoholis* | JG-1b | GCA_001541205.1 | McMurdo Dry Valleys, Antarctica | Permafrost |
| Terrestrial | *toruloides* | NBRC 0880 | ASM98887v2 | Japan | Soil |
| Terrestrial | *Rhodotorula. sp* | CCFEE 5036 | ASM505987v1 | Edmonson Point, Antarctica | Dry Soil |

**Table S2**. Bacterial and archaeal genomes selected for comparison to *Pelagibacter ubique* HTCC1062 and *Nitrosopumilus maritimus* SCM1.

| **Species** | **Strain** | **NCBI Accession No.** | **Genome Size (bp)** |
| --- | --- | --- | --- |
|  |  |  |  |
| *Pelagibacter ubique* | HTCC1062 | ASM1234v1 | 1,308,759 |
|  |  |  |  |
| *Rickettsia tillamookensis* | Tillamook 23 | ASM1674379v2 | 1,438,972 |
| *Rickettsia monacensis* | IrR/Munich | GCF_000499665.2 | 1,353,450 |
| *Rickettsia bellii* | An04 | ASM207831v1 | 1,579,798 |
| *Rickettsia slovaca* | 13-B | ASM23784v1 | 1,275,089 |
| *Rickettsia asiatica* | Maytaro1284 | ASM798942v1 | 1,419,085 |
|  |  |  |  |
| *Nitrosopumilus maritimus* | SCM1 | ASM1846v1 | 1,645,259 |
|  |  |  |  |
| *Nitrososphaera viennensis* | EN76 | ASM69878v1 | 2,527,938 |
| *Nitrososphaera evergladensis* | SR1 | ASM73028v1 | 2,954,373 |
| *Nitrosarchaeum koreense* | MY1 | ASM22017v1 | 1,607,695 |
| *Nitrososphaera gargensis* | Ga9.2 | ASM30315v1 | 2,833,868 |
| *Nitrosocosmicus oleophilus* | MY3 | ASM80220v2 | 3,430,706 |

**Table S3**. Eukaryotic clusters of orthologous groups (KOGs) assigned to each functional category for fifteen *Rhodotorula* species.

|  | **KOG Category** | *R. sphaerocarpa* ETNP2018 | *R. sphaerocarpa* GDMCC 60679 | *R. mucilaginosa* CYJ03 | *R. diobovata* 08-225 | *R. paludigena* P4R5 | *R. taiwanensis* MD1149 | *Rhodotorula sp.* CCFEE5036 | *R. frigidialcoholis* JG-1b | *R. mucilaginosa* F6_4S_B_2B | *R. mucilaginosa* B1 | *R. toruloides* NBRC 0880 | *R. graminis* WP1 | *R. kratochvilovae* Y14 | *R. kratochvilovae* Y25235 | *R. glutinis* ZHK |
| --- | --- | --- | --- | --- | --- | --- | --- | --- | --- | --- | --- | --- | --- | --- | --- | --- |
| **A** | **Carbohydrate Transport and Metabolism** | 257 | 257 | 294 | 318 | 343 | 295 | 281 | 282 | 277 | 296 | 324 | 292 | 339 | 366 | 322 |
| **B** | **Energy Production and Conversion** | 216 | 211 | 216 | 232 | 225 | 216 | 218 | 209 | 210 | 220 | 238 | 219 | 230 | 241 | 234 |
| **C** | **Lipid Transport and Metabolism** | 217 | 221 | 232 | 246 | 244 | 224 | 225 | 225 | 221 | 236 | 252 | 238 | 247 | 266 | 238 |
| **D** | **Secondary Metabolites Transport and Metabolism** | 152 | 143 | 164 | 176 | 207 | 166 | 165 | 163 | 156 | 161 | 198 | 171 | 204 | 213 | 179 |
| **E** | **Amino Acid Transport and Metabolism** | 277 | 280 | 293 | 312 | 333 | 286 | 274 | 281 | 280 | 290 | 317 | 309 | 325 | 339 | 313 |
| **F** | **Nucleotide Transport and Metabolism** | 83 | 81 | 86 | 91 | 91 | 86 | 79 | 74 | 81 | 85 | 89 | 90 | 94 | 102 | 100 |
| **G** | **Coenzyme Transport and Metabolism** | 126 | 124 | 133 | 142 | 144 | 128 | 125 | 126 | 126 | 137 | 143 | 137 | 143 | 149 | 138 |
| **H** | **Inorganic Ion Transport and Metabolism** | 150 | 150 | 144 | 153 | 170 | 154 | 136 | 142 | 132 | 144 | 179 | 145 | 169 | 169 | 155 |
| **I** | **Chromatin Structure and Dynamics** | 96 | 99 | 95 | 103 | 107 | 101 | 97 | 89 | 90 | 104 | 116 | 99 | 104 | 114 | 106 |
| **J** | **Translation/Ribosomal Structure and Biogenesis** | 325 | 329 | 333 | 329 | 336 | 333 | 313 | 324 | 329 | 340 | 358 | 323 | 338 | 368 | 330 |
| **K** | **Transcription** | 270 | 263 | 274 | 270 | 297 | 264 | 266 | 245 | 258 | 276 | 309 | 252 | 291 | 297 | 286 |
| **L** | **Replication, Recombination, and Repair** | 200 | 204 | 203 | 205 | 201 | 190 | 193 | 181 | 192 | 202 | 221 | 185 | 214 | 226 | 203 |
| **M** | **RNA Processing and Modification** | 275 | 281 | 277 | 279 | 279 | 282 | 264 | 243 | 274 | 274 | 284 | 264 | 276 | 299 | 277 |
| **N** | **Post-Translational Modification/Protein Turnover/Chaperones** | 430 | 433 | 442 | 445 | 459 | 425 | 421 | 417 | 418 | 442 | 491 | 437 | 465 | 478 | 457 |
| **O** | **Defense Mechanisms** | 36 | 36 | 39 | 36 | 42 | 39 | 35 | 37 | 37 | 38 | 44 | 32 | 39 | 38 | 37 |
| **P** | **Signal Transduction Mechanisms** | 328 | 319 | 343 | 349 | 350 | 330 | 318 | 306 | 322 | 338 | 362 | 313 | 358 | 372 | 358 |
| **Q** | **Cell Cycle Control/Cell Division/Chromosome Partitioning** | 151 | 154 | 155 | 159 | 161 | 155 | 143 | 144 | 150 | 159 | 164 | 149 | 161 | 169 | 170 |
| **R** | **Cytoskeleton** | 109 | 108 | 112 | 115 | 116 | 110 | 104 | 101 | 104 | 112 | 115 | 107 | 113 | 118 | 118 |
| **S** | **Nuclear Structure** | 28 | 27 | 28 | 25 | 28 | 28 | 26 | 23 | 27 | 30 | 29 | 24 | 30 | 29 | 30 |
| **T** | **Intracellular Trafficking, Secretion, and Vesicular Transport** | 347 | 342 | 355 | 348 | 367 | 355 | 337 | 326 | 330 | 359 | 373 | 336 | 375 | 394 | 363 |
| **U** | **Cell Wall/Membrane/Envelope Biogenesis** | 58 | 59 | 61 | 65 | 63 | 66 | 62 | 61 | 56 | 63 | 77 | 60 | 67 | 70 | 59 |
| **V** | **Extracellular Structures** | 6 | 6 | 7 | 6 | 7 | 7 | 8 | 7 | 7 | 7 | 8 | 6 | 8 | 7 | 6 |
| **W** | **Cell Motility** | 6 | 6 | 5 | 4 | 7 | 6 | 5 | 5 | 3 | 6 | 7 | 7 | 7 | 8 | 7 |
| **X** | **Function Unknown** | 1181 | 1167 | 1238 | 1312 | 1336 | 1235 | 1164 | 1105 | 1163 | 1230 | 1432 | 1226 | 1437 | 1461 | 1349 |
|  | **Total** | **5324** | **5300** | **5529** | **5720** | **5913** | **5481** | **5259** | **5116** | **5243** | **5549** | **6130** | **5421** | **6034** | **6293** | **5835** |

**Table S4**. Statistical analysis of the depletion of specific KOG categories in *Rhodotorula sphaerocarpa* ETNP2018, performed via one sample T-test. All tests were performed using a 95% confidence interval and the average of all fifteen representative *Rhodotorula* strains.

| **KOG Category** | **Number present in *R. sphaerocarpa* ETNP2018** | **Average of the 15 *Rhodotorula* strains** | **p-value** |
| --- | --- | --- | --- |
| All KOGs | 5324 | 5609.8 | 0.00411 |
| Translation/Rib Genesis | 325 | 333.9 | 0.01253 |
| Transcription | 270 | 274.5 | 0.17494 |
| Carbohydrate | 257 | 302.9 | 0.00003 |
| Lipid | 217 | 235.5 | 0.00007 |
| Secondary Metabolites | 152 | 174.5 | 0.00057 |
| Amino Acid | 277 | 300.6 | 0.00044 |
| Coenzyme | 126 | 134.7 | 0.00062 |

**Table S5**. CAZyme families present in 15 representative *Rhodotorula* strains determined using dbCAN2. The carbohydrate binding module domains (CBM) column only includes genes containing both glycoside hydrolase (GH) and carbohydrate binding module domains (CBM).

| ***Rhodotorula* Species** | **Strain** | **GH** | **GT** | **AA** | **CBM** | **CE** | **PL** | **Total** |
| --- | --- | --- | --- | --- | --- | --- | --- | --- |
| *sphaerocarpa* | ETNP2018 | 56 | 84 | 17 | 3 | 8 | 4 | 172 |
| *sphaerocarpa* | GDMCC 60679 | 53 | 81 | 16 | 3 | 8 | 4 | 165 |
| *diobovata* | 08-225 | 73 | 96 | 22 | 4 | 13 | 6 | 214 |
| *paludigena* | P4R5 | 75 | 100 | 25 | 3 | 14 | 5 | 222 |
| *glutinis* | ZHK | 76 | 98 | 22 | 3 | 14 | 6 | 219 |
| *taiwanensis* | MD1149 | 67 | 97 | 16 | 3 | 7 | 4 | 194 |
| *graminis* | WP1 | 66 | 91 | 19 | 3 | 13 | 5 | 197 |
| *kratochvilovae* | YM25235 | 65 | 95 | 16 | 3 | 9 | 4 | 192 |
| *kratochvilovae* | Y14 | 60 | 95 | 16 | 6 | 8 | 4 | 189 |
| *toruloides* | NBRC 0880 | 76 | 93 | 24 | 3 | 13 | 4 | 213 |
| *mucilaginosa* | CYJ03 | 56 | 87 | 16 | 2 | 9 | 3 | 173 |
| *mucilaginosa* | F6_4S_B_2B | 76 | 98 | 22 | 3 | 15 | 6 | 220 |
| *mucilaginosa* | B1 | 65 | 95 | 16 | 3 | 9 | 4 | 192 |
| *frigidialcoholis* | JG-1b | 57 | 90 | 15 | 3 | 7 | 2 | 174 |
| *Rhodotorula. sp* | CCFEE 5036 | 61 | 88 | 13 | 3 | 9 | 4 | 178 |

**Table S6**. All CAZymes (including their described functions) present in 15 representative *Rhodotorula* strains.

| CAZyme Family | Described Function | *R. sphaerocarpa* ETNP2018 | *R. sphaerocarpa* GDMCC 60679 | *R. mucilaginosa* CYJ03 | *R. diobovata* 08-225 | *R. paludigena* P4R5 | *R. taiwanensis* MD1149 | *Rhodotorula. sp.* CCFEE5036 | *R. frigidialcoholis* JG-1b | *R. mucilaginosa* F6_4S_B_2B | *R. mucilaginosa* B1 | *R. toruloides* NBRC 0880 | *R. graminis* WP1 | *R. kratochvilovae* Y14 | *R. kratochvilovae* Y25235 | *R. glutinis* ZHK |
| --- | --- | --- | --- | --- | --- | --- | --- | --- | --- | --- | --- | --- | --- | --- | --- | --- |
| AA1 | laccase | 5 | 4 | 4 | 2 | 3 | 4 | 3 | 4 | 4 | 4 | 4 | 2 | 2 | 2 | 3 |
| AA11 | Cu-dependent lytic plysaccharide monooxygenases | 2 | 2 | 2 | 2 | 2 | 2 | 2 | 1 | 2 | 2 | 2 | 1 | 2 | 2 | 2 |
| AA14 | Cu-dependent lytic plysaccharide monooxygenases | 2 | 2 | 2 | 3 | 3 | 2 | 1 | 2 | 2 | 2 | 3 | 2 | 4 | 4 | 3 |
| AA2 | Mn peroxidase | 2 | 2 | 2 | 2 | 2 | 2 | 1 | 2 | 2 | 2 | 2 | 2 | 2 | 2 | 2 |
| AA3 | Cellobiose dehydrogenase | 2 | 2 | 2 | 5 | 5 | 2 | 2 | 2 | 2 | 2 | 4 | 5 | 5 | 5 | 4 |
| AA4 | vanillyl-alcohol oxidase | 0 | 0 | 0 | 0 | 2 | 0 | 0 | 0 | 0 | 0 | 2 | 0 | 2 | 2 | 0 |
| AA5 | galactose oxidase | 2 | 2 | 2 | 3 | 3 | 2 | 2 | 2 | 2 | 2 | 2 | 3 | 4 | 4 | 3 |
| AA6 | 1,4-benzoquinone reductase | 1 | 1 | 1 | 1 | 1 | 1 | 1 | 1 | 1 | 1 | 0 | 1 | 1 | 1 | 1 |
| AA7 | glucooligosaccharide oxidase | 1 | 1 | 0 | 1 | 1 | 2 | 0 | 0 | 0 | 0 | 0 | 1 | 1 | 0 | 1 |
| AA8 | Iron reductase domain | 0 | 0 | 0 | 1 | 0 | 0 | 0 | 0 | 0 | 0 | 1 | 0 | 1 | 0 | 0 |
| AA9 | Cu-dependent lytic plysaccharide monooxygenases | 0 | 0 | 0 | 1 | 2 | 0 | 0 | 0 | 0 | 0 | 1 | 1 | 2 | 2 | 1 |
| CBM1 | Cellulose | 2 | 2 | 2 | 1 | 2 | 2 | 2 | 2 | 2 | 2 | 2 | 1 | 1 | 1 | 1 |
| CBM21 | Granular starch-binding | 1 | 1 | 1 | 1 | 1 | 1 | 1 | 1 | 1 | 1 | 0 | 1 | 1 | 3 | 1 |
| CE1 | acetyl xylan esterase | 1 | 1 | 1 | 1 | 2 | 1 | 1 | 1 | 1 | 1 | 1 | 1 | 1 | 1 | 1 |
| CE10 | arylesterase | 14 | 13 | 17 | 14 | 18 | 14 | 15 | 14 | 13 | 15 | 17 | 14 | 13 | 11 | 14 |
| CE16 | acetylesterase | 2 | 2 | 4 | 6 | 7 | 4 | 4 | 3 | 4 | 4 | 6 | 5 | 7 | 8 | 6 |
| CE4 | acetyl xylan esterase | 5 | 5 | 6 | 5 | 4 | 4 | 7 | 5 | 7 | 7 | 6 | 6 | 6 | 4 | 6 |
| CE5 | acetyl xylan esterase | 0 | 0 | 0 | 2 | 1 | 0 | 0 | 0 | 0 | 0 | 1 | 2 | 1 | 1 | 2 |
| GH105 | unsaturated rhamnogalacturonyl hydrolase | 2 | 2 | 2 | 2 | 2 | 2 | 1 | 2 | 1 | 1 | 2 | 2 | 0 | 0 | 1 |
| GH128 | β-1,3-glucanase | 2 | 1 | 3 | 2 | 3 | 2 | 3 | 2 | 1 | 3 | 2 | 1 | 3 | 3 | 2 |
| GH13 | a-amylase | 2 | 2 | 4 | 3 | 3 | 6 | 3 | 4 | 4 | 4 | 3 | 2 | 3 | 3 | 3 |
| GH133 | amylo-a-1,6-glucosidase | 1 | 1 | 1 | 0 | 1 | 1 | 1 | 1 | 1 | 1 | 0 | 1 | 0 | 0 | 0 |
| GH135 | α-1,4-galactosaminogalactan hydrolase | 1 | 1 | 0 | 1 | 1 | 1 | 0 | 0 | 0 | 0 | 1 | 2 | 1 | 0 | 2 |
| GH15 | glucoamylase | 2 | 2 | 2 | 2 | 2 | 2 | 2 | 2 | 2 | 2 | 2 | 2 | 3 | 3 | 2 |
| GH152 | β-1,3-glucanase | 3 | 2 | 3 | 4 | 4 | 4 | 3 | 2 | 3 | 3 | 4 | 4 | 4 | 2 | 4 |
| GH16 | xyloglucanase | 6 | 6 | 6 | 9 | 8 | 9 | 5 | 3 | 4 | 5 | 4 | 10 | 6 | 6 | 10 |
| GH17 | glucan ando-1.3-β-glucosidase | 1 | 1 | 1 | 1 | 1 | 1 | 1 | 1 | 1 | 1 | 1 | 1 | 1 | 0 | 1 |
| GH18 | chitinase | 3 | 3 | 4 | 6 | 4 | 4 | 4 | 4 | 4 | 4 | 7 | 3 | 5 | 8 | 6 |
| GH2 | β-galactosidase | 0 | 0 | 1 | 1 | 1 | 1 | 1 | 1 | 1 | 1 | 1 | 1 | 1 | 1 | 1 |
| GH20 | β-hexosaminidase | 1 | 1 | 2 | 2 | 2 | 1 | 2 | 2 | 1 | 2 | 2 | 1 | 2 | 2 | 1 |
| GH26 | β-mannanase | 0 | 0 | 1 | 1 | 0 | 1 | 1 | 1 | 0 | 1 | 0 | 0 | 1 | 0 | 0 |
| GH28 | Polygalacturonase | 0 | 0 | 0 | 1 | 0 | 0 | 0 | 0 | 0 | 0 | 1 | 1 | 2 | 2 | 0 |
| GH3 | β-glucosidase | 3 | 3 | 4 | 5 | 5 | 4 | 4 | 3 | 4 | 4 | 5 | 4 | 4 | 3 | 4 |
| GH31 | a-glucosidase | 1 | 1 | 1 | 1 | 1 | 1 | 1 | 1 | 1 | 1 | 1 | 1 | 1 | 1 | 1 |
| GH32 | Invertase | 1 | 1 | 1 | 1 | 1 | 2 | 1 | 1 | 1 | 1 | 1 | 1 | 1 | 1 | 1 |
| GH37 | α,α-trehalase | 1 | 1 | 1 | 1 | 1 | 1 | 1 | 1 | 1 | 1 | 1 | 1 | 1 | 1 | 1 |
| GH38 | α-mannosidase | 1 | 1 | 1 | 1 | 1 | 1 | 1 | 1 | 1 | 1 | 1 | 1 | 1 | 1 | 1 |
| GH43 | β-xylosidase | 0 | 0 | 0 | 1 | 1 | 0 | 0 | 0 | 0 | 0 | 0 | 1 | 2 | 2 | 1 |
| GH47 | α-mannosidase | 3 | 3 | 3 | 3 | 3 | 3 | 2 | 3 | 3 | 3 | 3 | 3 | 4 | 2 | 3 |
| GH5 | cellulase | 9 | 9 | 10 | 11 | 13 | 9 | 9 | 10 | 9 | 10 | 12 | 11 | 14 | 14 | 13 |
| GH63 | α-glucosidase | 0 | 0 | 0 | 0 | 0 | 0 | 0 | 0 | 0 | 0 | 0 | 0 | 0 | 1 | 0 |
| GH65 | α,α-trehalase | 1 | 1 | 1 | 1 | 1 | 1 | 1 | 1 | 1 | 1 | 1 | 1 | 1 | 1 | 1 |
| GH71 | α-1,3-glucanase | 1 | 1 | 0 | 1 | 2 | 0 | 0 | 0 | 0 | 0 | 1 | 1 | 1 | 1 | 1 |
| GH75 | chitosanase | 0 | 0 | 0 | 1 | 1 | 0 | 0 | 0 | 0 | 0 | 1 | 1 | 1 | 1 | 1 |
| GH76 | α-1,6-mannanase | 0 | 0 | 1 | 1 | 1 | 1 | 1 | 1 | 1 | 1 | 1 | 1 | 1 | 1 | 1 |
| GH78 | α-L-rhamnosidase | 0 | 0 | 1 | 0 | 1 | 0 | 1 | 1 | 1 | 1 | 1 | 1 | 1 | 1 | 0 |
| GH85 | endo-β-N-acetylglucosaminidase | 1 | 1 | 1 | 1 | 1 | 1 | 1 | 1 | 1 | 1 | 1 | 0 | 1 | 0 | 1 |
| GH88 | β-glucuronyl hydrolase | 0 | 0 | 1 | 0 | 0 | 1 | 1 | 1 | 1 | 1 | 1 | 0 | 1 | 0 | 0 |
| PL1 | Pectate lyase | 0 | 0 | 0 | 1 | 1 | 0 | 0 | 0 | 0 | 0 | 0 | 1 | 2 | 3 | 1 |
| PL14 | poly(β-mannuronate) lyase | 3 | 3 | 3 | 3 | 3 | 3 | 3 | 1 | 3 | 3 | 3 | 3 | 5 | 6 | 4 |
| PL35 | chondroitin lyase | 1 | 1 | 1 | 1 | 1 | 1 | 1 | 1 | 0 | 1 | 1 | 1 | 1 | 1 | 1 |
| PL36 | poly(β-mannuronate) lyase | 0 | 0 | 1 | 1 | 1 | 0 | 0 | 0 | 0 | 0 | 1 | 1 | 0 | 0 | 1 |
| PL4 | rhamnogalacturonan endolyase | 0 | 0 | 0 | 0 | 0 | 0 | 0 | 0 | 0 | 0 | 0 | 0 | 1 | 1 | 0 |


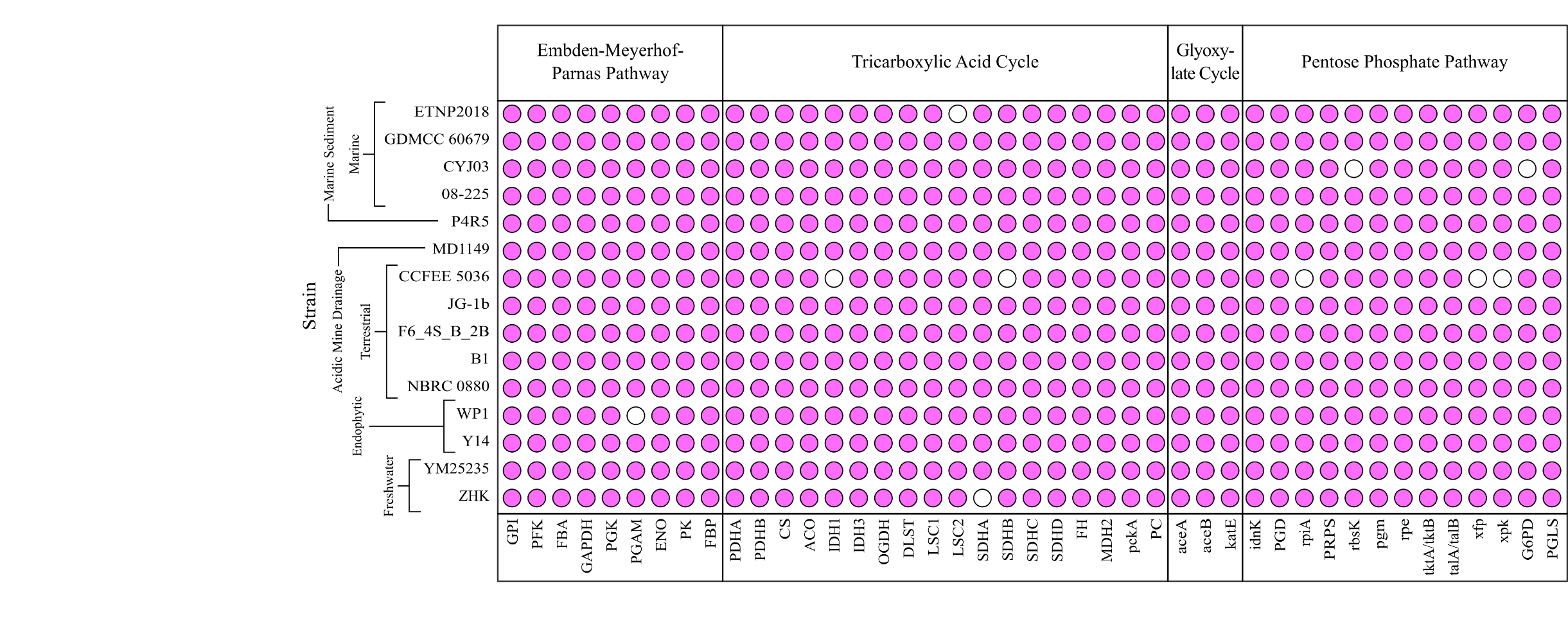


**Figure S1**. A heatmap showing highly conserved carbohydrate metabolism pathways in the *Rhodotorula* genus. Genes present in the genome are represented by filled circles; genes absent in the genome are represented by empty circles. Abbreviations for the gene names are as follows: GPI, glucose-6-phosphate isomerase; PFK, 6-phosphofructokinase 1; FBA, fructose-bisphosphate aldolase, class II; GAPDH, glyceraldehyde 3-phosphate dehydrogenase; PGK, phosphoglycerate kinase; PGAM, 2,3-bisphosphoglycerate-dependent phosphoglycerate mutase; ENO, enolase; PK, pyruvate kinase; FBP, fructose-1,6-bisphosphatase I; PDHA, pyruvate dehydrogenase E1 component alpha subunit; PDHB, pyruvate dehydrogenase E1 component beta subunit; CS, citrate synthase; ACO, aconitate hydratase; IDH1, isocitrate dehydrogenase; IDH3, isocitrate dehydrogenase (NAD+); OGDH, 2-oxoglutarate dehydrogenase; DLST, dihydrolipoamide succinyltransferase; LSC1, succinyl-CoA synthetase alpha subunit; LSC2, succinyl-CoA synthetase beta subunit; SDHA, succinate dehydrogenase flavoprotein subunit; SDHB, succinate dehydrogenase iron-sulfur subunit; SDHC, succinate dehydrogenase cytochrome B560 subunit; SDHD, succinate dehydrogenase membrane anchor subunit; FH, fumarate hydratase; MDH2, malate dehydrogenase; pckA, phophoenolpyruvate carboxykinase (ATP); PC, pyruvate carboxylase; aceA, isocitrate lyase; aceB, malate synthase; katE, catalase; idnK, gluconokinase; PGD, 6-phosphogluconate dehydrogenase; rpiA, ribose 5-phosphate pyrophosphokinase; PRPS, ribose-phosphate pyrophosphokinase; rbsK, ribokinase; pgm, phosphoglucomutase; rpe, ribulose-phosphate 3-epimerase; tktA/tktB, transketolase; talA/talB, transaldolase; xfp, xylulose-5-phosphate/fructose-6-phosphate phosphoketolase; xpk, fructose-6-phosphate phophoketolase; G6PD, glucose-6-phosphate 1-dehydrogenase; PGLS, 6-phosphogluconolactonase.


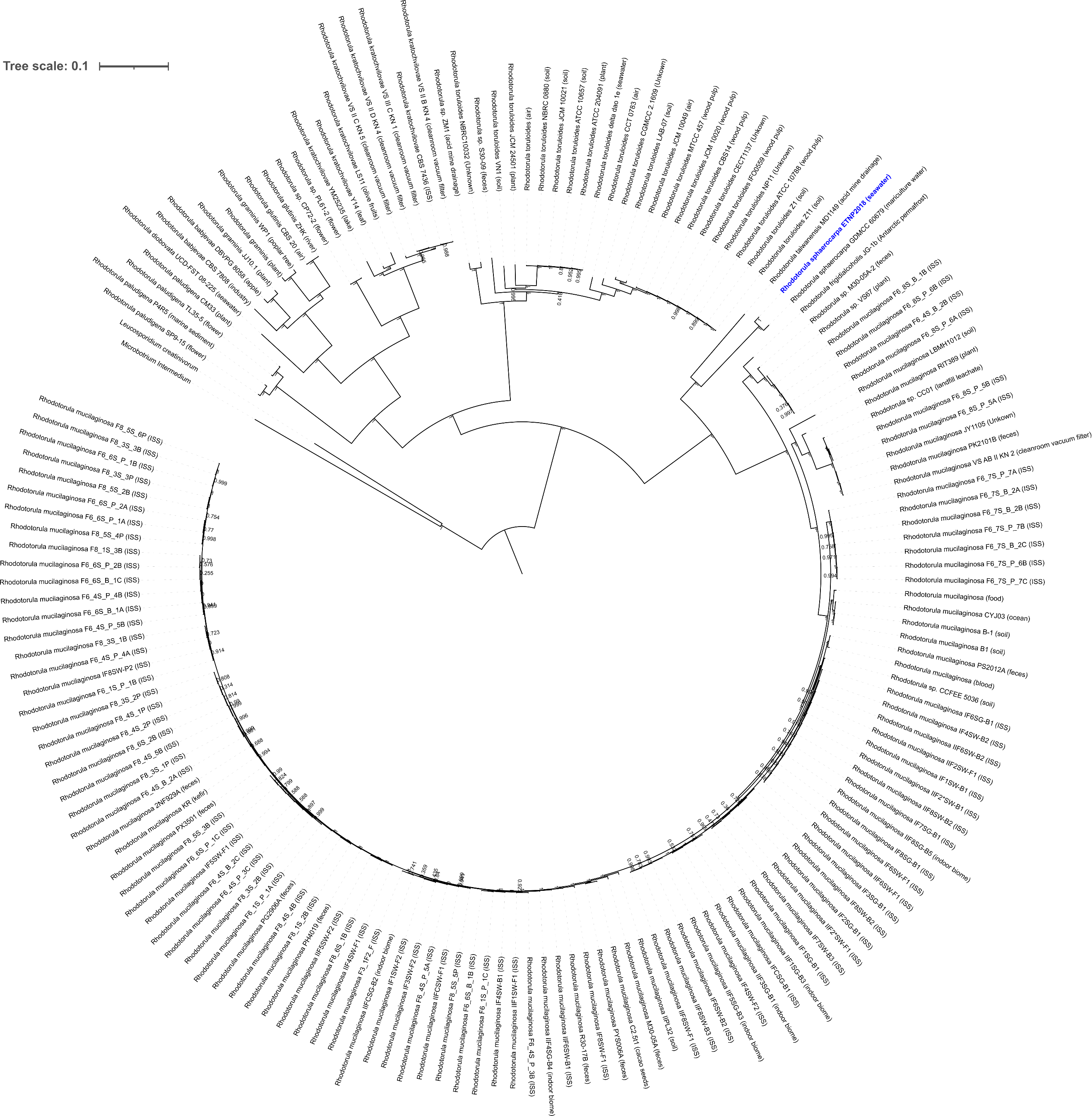


**Figure S2**. A phylogenomic tree constructed using single-copy orthologues shared amongst 168 representative *Rhodotorula* strains (as well as two outgroup species, *Microbotrium intermedium* GCA 900096595.1 and *Leucosporidium creatinivorum* GCA 002105055.1). 983 single-copy orthologs were retrieved for comparison using BUSCO v5.4.4. The isolation source is denoted in the parentheses after the strain names (“ISS” = International Space Station). Bootstrap values (n = 1000) are displayed at each node. *R. sphaerocarpa* ETNP2018 is highlighted by a bold blue font.


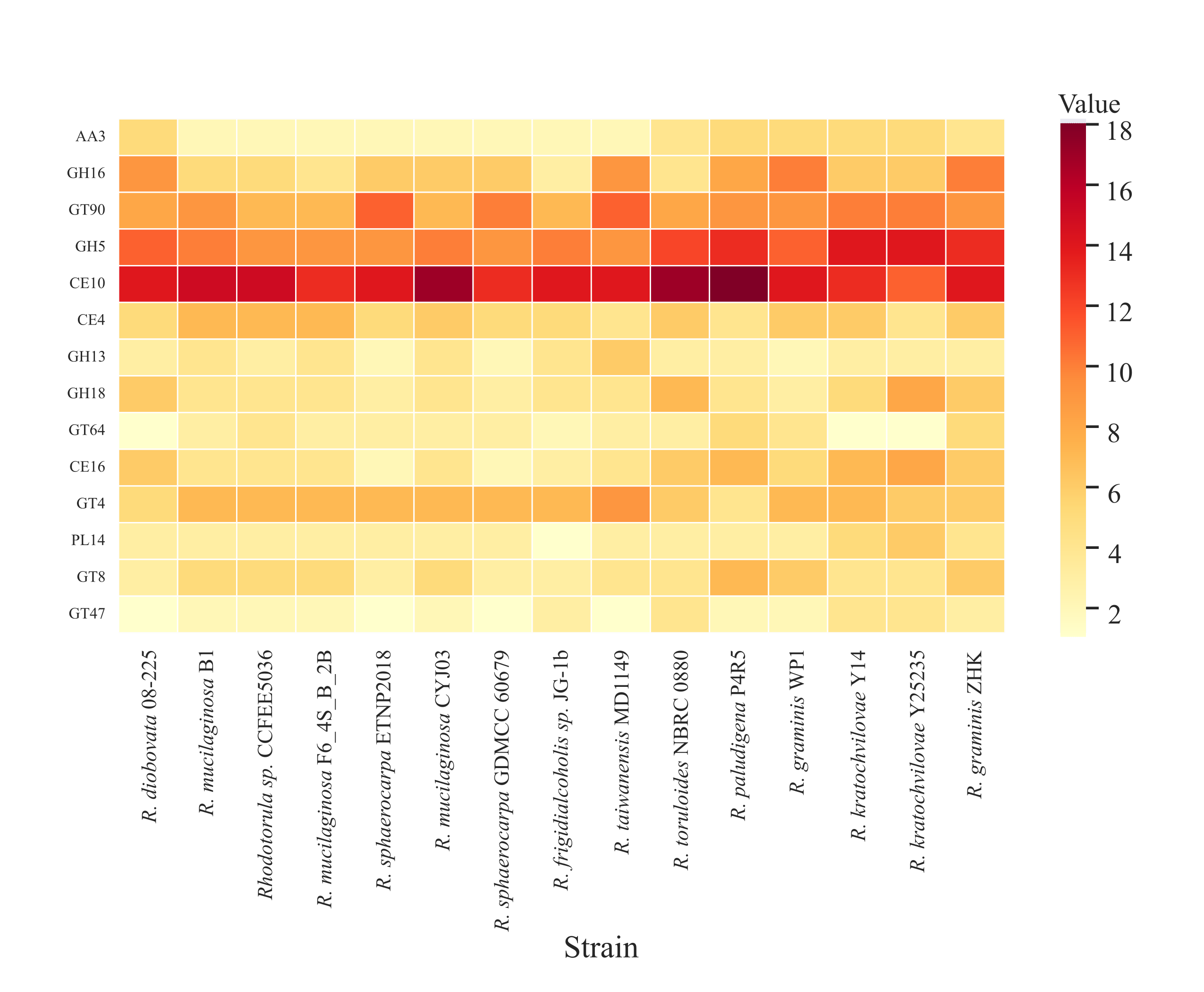


**Figure S3**. Heatmap showing the number of major CAZymes present in the *Rhodotorula* genus.
